# The prognostic significance of primary tumor size in squamous cell carcinoma of the penis

**DOI:** 10.1007/s12672-021-00416-7

**Published:** 2021-07-19

**Authors:** Kai Li, Guang Wu, Caibin Fan, Hexing Yuan

**Affiliations:** 1 Department of Urology, The Affiliated Suzhou Hospital of Nanjing Medical University, Suzhou Municipal Hospital, Gusu School, Nanjing Medical University, 26 Daoqian Road, Suzhou, 215000 Jiangsu Province People’s Republic of China; 2 Department of Urology, First Affiliated Hospital of Soochow University, 188 Shizi Road, Suzhou, 215000 Jiangsu Province People’s Republic of China

**Keywords:** Tumor size, Prognostic value, Squamous cell carcinoma of the penis, Overall survival, Penile carcinoma-specific survival

## Abstract

**Background:**

To evaluate the association of primary tumor size with clinicopathologic characteristics and survival of patients with squamous cell carcinoma of the penis (SCCP).

**Methods:**

This study analyzed the data of 1001 patients with SCCP, obtained from the National Cancer Institute Surveillance, Epidemiology, and End Results (SEER) database between 2010 and 2014. The Kaplan–Meier method and the Cox proportional hazards regression model were used to analyze the effects of primary tumor size on overall survival (OS) and penile carcinoma-specific survival (PCSS).

**Results:**

Advanced T stage (P < 0.001), lymph node metastasis (P < 0.001) and distant metastasis (P = 0.001) were more frequently associated with SCCP patients with tumor size ≥ 3 cm than those with tumor size  < 3 cm. In Kaplan–Meier analyses, the patients with large tumors (≥ 3 cm) exhibited an inferior OS and PCSS than those with small tumors (< 3 cm). Moreover, tumor size was identified to be an independent prognostic factor for OS [hazard ratio (HR) 1.665, P < 0.001] and PCSS (HR 2.076, P = 0.003) of patients with SCCP in multivariate analyses.

**Conclusions:**

Large tumor size is associated with adverse clinicopathological characteristics of patients with SCCP. Besides, tumor size represents an independent prognostic factor for OS and PCSS. Therefore, clinical assessment of tumor size as a crucial prognostic factor might be highly beneficial for early intervention in patients with SCCP.

## Introduction

**Fig. 1:**
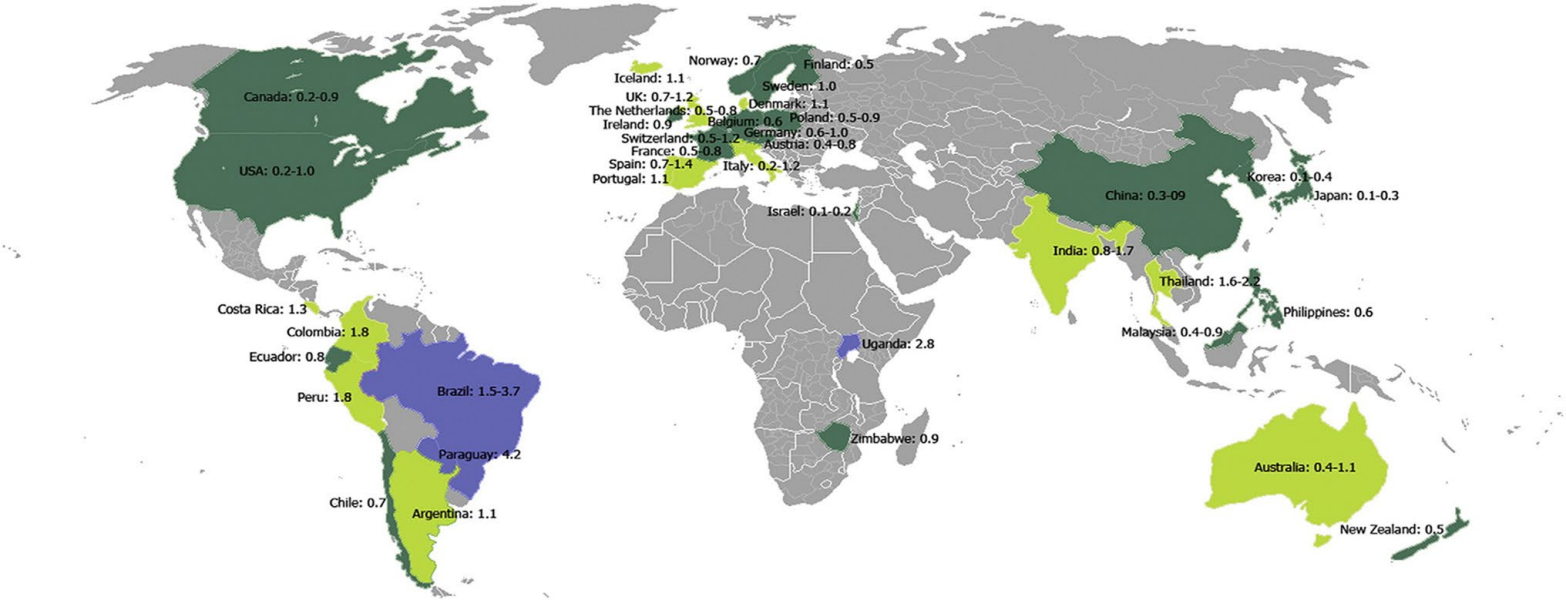
The incidence of penile cancer in various regions of the world. Blue areas represent high incidence; light green areas represent moderate morbidity; dark green areas represent low morbidity (reported incidence in brackets). Reused by permission from: Chaux A and Cubilla AL [1]

Penile carcinoma is a relatively rare malignancy, with incidence varying from country to country (Fig. 1) [1, 2]. The etiology of penile carcinoma is multifactorial; however, the incidence of penile carcinoma is particularly high in regions with a high prevalence of human papillomavirus (HPV), and approximately one-third of cases can be attributed to HPV infection [3]. Besides, numerous other etiologic factors including phimosis, chronic inflammation, poor hygiene, balanitis xerotica obliterans, penile trauma, human immunodeficiency virus and tobacco consumption have been identified to increase the risk of developing penile carcinoma [4, 5].

Squamous cell carcinoma of the penis (SCCP) is the predominant histological type of penile carcinomas [6, 7]. It usually occurs in men between the ages of 50 and 70 years [8]. The majority of lesions occur on the glans (48%), followed by the prepuce (21%), both glans and prepuce (15%), coronal sulcus (6%), and shaft (< 2%) [9]. Although the majority of cases are limited when diagnosed, approximately 25% of SCCP patients may have regional (inguinal and/or pelvic) lymph node metastasis and nearly 4% may have a distant disease [10]. Once metastasis occurs, the prognosis could be very poor and the mortality rate increases [3]. Furthermore, Novara et al. reported that men with SCCP, if remain untreated, usually die within 2 years post-diagnosis, due to uncontrollable locoregional lesion or distant metastases [4]. Currently, organ preservation approaches remain the treatment of choice for most patients with early tumors. However, for invasive tumors, a partial or radical penectomy is the oncological gold standard therapy [3, 11].

An increasing number of studies have been conducted to identify significant prognostic factors for SCCP. Wiechno et al. reported that the presence of metastases in the lymph nodes significantly predicted a worse prognosis for patients with SCC of the penis [12]. Sanchez et al. found that histologic subtypes of SCCP played a crucial role in determining risk for patients’ survival [13]. Besides, several other prognostic indicators, including histological grade, lymphovascular embolization and primary tumor stage have been studied [14, 15]. Nevertheless, the prognostic value of tumor size in SCCP remains elusive. Moreover, the tumor size of SCCP is not considered as an important criterion for classification in the Tumor-Node-Metastasis (TNM) staging system, developed by the Union for International Cancer Control (UICC). Using the Surveillance, Epidemiology, and End Results (SEER)-registered database, the present study aimed to evaluate the relationship between primary tumor size and clinicopathologic characteristics and survival of men with SCCP.

## Methods

### Study population

**Fig. 2:**
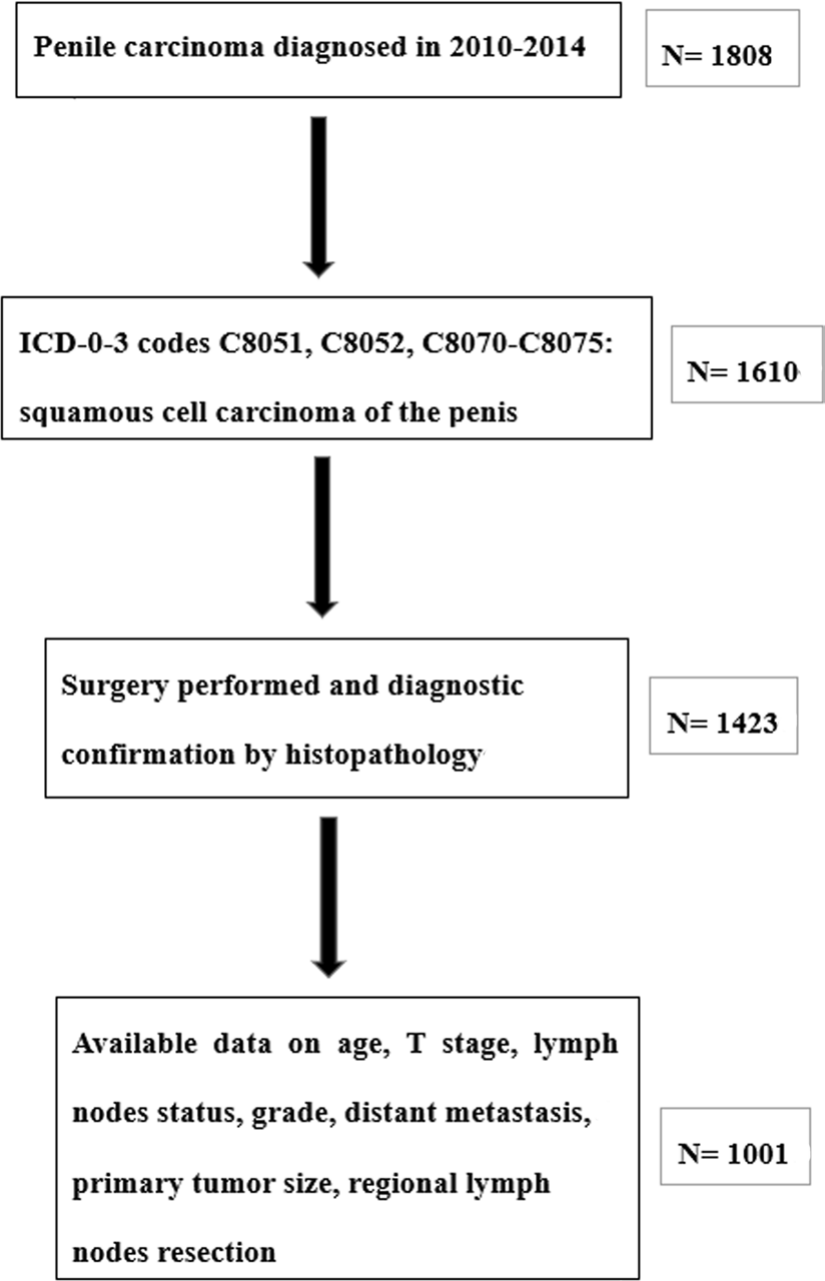
Selection procedure

We extracted the clinical data of patients with SCCP diagnosed between 2010 and 2014 from the SEER database. The inclusion criteria were as follows: (1) Penile carcinoma with codes 8051, 8052 and 8070–8075 were considered as SCCP as defined by International Classification of Diseases for Oncology, 3rd Edition (ICD-O-3) [16]; (2) Patients underwent surgery and had histopathologically confirmed diagnosis of SCCP; and (3) Patients with available data on age, T stage, lymph nodes status, grade, distant metastasis, regional lymph nodes removed, surgery and tumor size. The selection procedure is showed in Fig. 2. Ultimately, a total of 1001 eligible patients were included.

As the data were de-identified, this study was considered exempt from approval by the Institutional Review Board. Also, the requirement for informed consent from patients was considered waived [17].

### Study variables

The primary endpoints of the study were overall survival (OS) and penile carcinoma-specific survival (PCSS). OS was defined as the time period from the date of SCCP diagnosis to the date of death from any cause or the date of the last follow-up [7]. PCSS was defined as the time interval from the date of diagnosis to the date of death from SCCP or the last follow-up date for censoring [7]. Covariates for each case included age, clinical and histological characteristics (T stage, lymph nodes status, grade, distant metastasis, regional lymph nodes removed, surgery, and tumor size). A previous study reported that the 5-year cure rates of patients with penile cancer lesions more than 3 cm was 50 per cent, while patients with lesions smaller than 3 cm had higher the 5-year cure rates [18]. So, according to these findings, 3 cm was determined as the cut-off value for tumor size in this study. TNM stages were determined according to the American Joint Committee on Cancer (AJCC) 7th edition staging system. The survival cut-off date was December 31, 2014 in this study. The cause of death was determined from the death certificate.

### Statistical analysis

The association between categorical variables was determined using the chi-square test. The Kaplan–Meier method was used to calculate survival probabilities, and the differences were assessed with the log-rank test. Significantly associated variables identified by Kaplan–Meier analyses were then entered into a Cox proportional hazards regression model for multivariate analysis, yielding hazard ratios (HR) and 95% confidence intervals (CI). Two-sided p-values of < 0.05 were considered statistically significant. All statistical analyses were performed using SPSS 17.0 (IBM Corporation, Armonk, NY, USA). All data were extracted using SEER*Stat Software version 8.3.5 (Information Management Sercives, Inc. Calverton, MD, USA).

## Results

Overall, a total of 1001 patients with SCCP meeting the eligibility criteria were included in the study. A total of 609 patients with SCCP who did not undergo surgery and did not provide relevant data on age, T stage, lymph nodes status, grade, distant metastasis, regional lymph nodes removed, surgery and tumor size were excluded. A total of 433 (43.3%) patients exhibited the tumor size < 3 cm, while 568 (56.7%) had tumor size ≥ 3 cm. In this study, the median follow-up period was 17 months (range, 0–59). A total of 276 (27.6%) patients with SCCP died in this study.

As presented in Table 1, we analyzed the association of tumor size with clinicopathological characteristics. The results revealed that advanced T stage (T1b–T4) (P < 0.001), lymph node metastasis (P < 0.001) and distant metastasis (P = 0.001) were more significantly associated with SCCP patients with tumor size ≥ 3 cm than those with tumor size < 3 cm. However, no statistically significant differences in age (P = 0.454) or grade (P = 0.068) were found between the two groups. Moreover, regional lymph nodes removed (P < 0.001) and radical surgery (P < 0.001) were more frequently performed in patients with tumor size ≥ 3 cm.

**Table 1:** Association of primary tumor size with clinicopathologic characteristics

N (%) variables	All patients	Tumor size < 3 cm	Tumor size ≥ 3 cm	P
No. of patients	1001	433	568	
Age				0.454
< 65	404 (40.4)	169 (41.8)	235 (58.2)	
≥ 65	597 (59.6)	264 (44.2)	333 (55.8)	
T stage				** < 0.001**
Tx–T1a	412 (41.2)	239 (58.0)	173 (42.0)	
T1b–T4	589 (58.8)	194 (32.9)	395 (67.1)	
Lymph nodes status				** < 0.001**
Nx–N0	822 (82.1)	391 (47.6)	431 (52.4)	
N1–N3	179 (17.9)	42 (23.5)	137 (76.5)	
Grade				0.068
G1 + G2	789 (78.8)	353 (44.7)	436 (55.3)	
G3 + G4	212 (21.2)	80 (37.7)	132 (62.3)	
Distant metastasis				**0.001**
M0	974 (97.3)	430 (44.1)	544 (55.9)	
M1	27 (2.7)	3 (11.1)	24 (88.9)	
Regional lymph nodes removed				** < 0.001**
No	778 (77.7)	366 (47.0)	412 (53.0)	
Yes	223 (22.3)	67 (30.0)	156 (70.0)	
Surgery				** < 0.001**
Non-radical surgery	964 (96.3)	428 (44.4)	536 (55.6)	
Radical surgery	37 (3.7)	5 (13.5)	32 (86.5)	

Significant values in bold

In Kaplan–Meier analyses, patients with tumor size ≥ 3 cm exhibited significantly lower OS and PCSS than those with tumor size < 3 cm (P < 0.001 for both, Table 2). We also found that T stage (P < 0.001 for both, Table 2), lymph nodes status (P < 0.001 for both, Table 2), grade (P < 0.001 for both, Table 2), distant metastasis (P < 0.001 for both, Table 2) and surgery (P = 0.004, P = 0.018, Table 2) were significantly associated with OS and PCSS. Besides, age (P < 0.001, Table 2) was significantly associated with OS, whereas regional lymph nodes removed (P = 0.046, Table 2) was significantly correlated with PCSS. Overall, these results indicated that age, T stage, lymph nodes status, grade, distant metastasis, surgery, tumor size, and regional lymph nodes removed were significant prognostics factors for poor survival. Subsequently, these variables were included in the multivariate analyses except surgery, after considering collinearity between the variables.

**Table 2:** Kaplan–Meier analyses predicting overall survival and penis cancer-specific survival in 1001 patients with SCCP

	Overall survival, %		Penis cancer-specific survival, %	
Variables	3-year probability (SEM)	*P*	3-year probability (SEM)	*P*
Age		** < 0.001**		0.677
< 65	73.6 (2.7)		79.0 (2.8)	
≥ 65	57.1 (2.6)		77.1 (2.9)	
T stage		** < 0.001**		** < 0.001**
Tx–T1a	74.0 (2.8)		90.0 (2.4)	
T1b–T4	56.0 (2.6)		69.1 (3.0)	
Lymph nodes status		** < 0.001**		** < 0.001**
Nx–N0	68.8 (2.1)		86.3 (1.9)	
N1–N3	39.8 (4.8)		43.4 (5.9)	
Grade		** < 0.001**		** < 0.001**
G1 + G2	66.5 (2.1)		81.3 (2.2)	
G3 + G4	51.8 (4.4)		64.4 (4.9)	
Distant metastasis		** < 0.001**		** < 0.001**
M0	64.8 (2.0)		80.2 (2.0)	
M1	21.0 (9.0)		22.9 (9.7)	
Regional lymph nodes removed		0.364		**0.046**
No	63.4 (2.2)		80.7 (2.2)	
Yes	64.0 (4.1)		70.7 (4.4)	
Surgery		**0.004**		**0.018**
Non-radical surgery	64.3 (2.0)		78.5 (2.1)	
Radical surgery	41.0 (11.6)		65.8 (10.7)	
Tumor size		** < 0.001**		** < 0.001**
< 3 cm	75.5 (2.6)		89.2 (2.5)	
≥ 3 cm	54.4 (2.7)		69.8 (2.9)	

*SCCP* squamous cell carcinoma of the penis, *SEM* standard error of mean

Significant values in bold

**Fig. 3:**
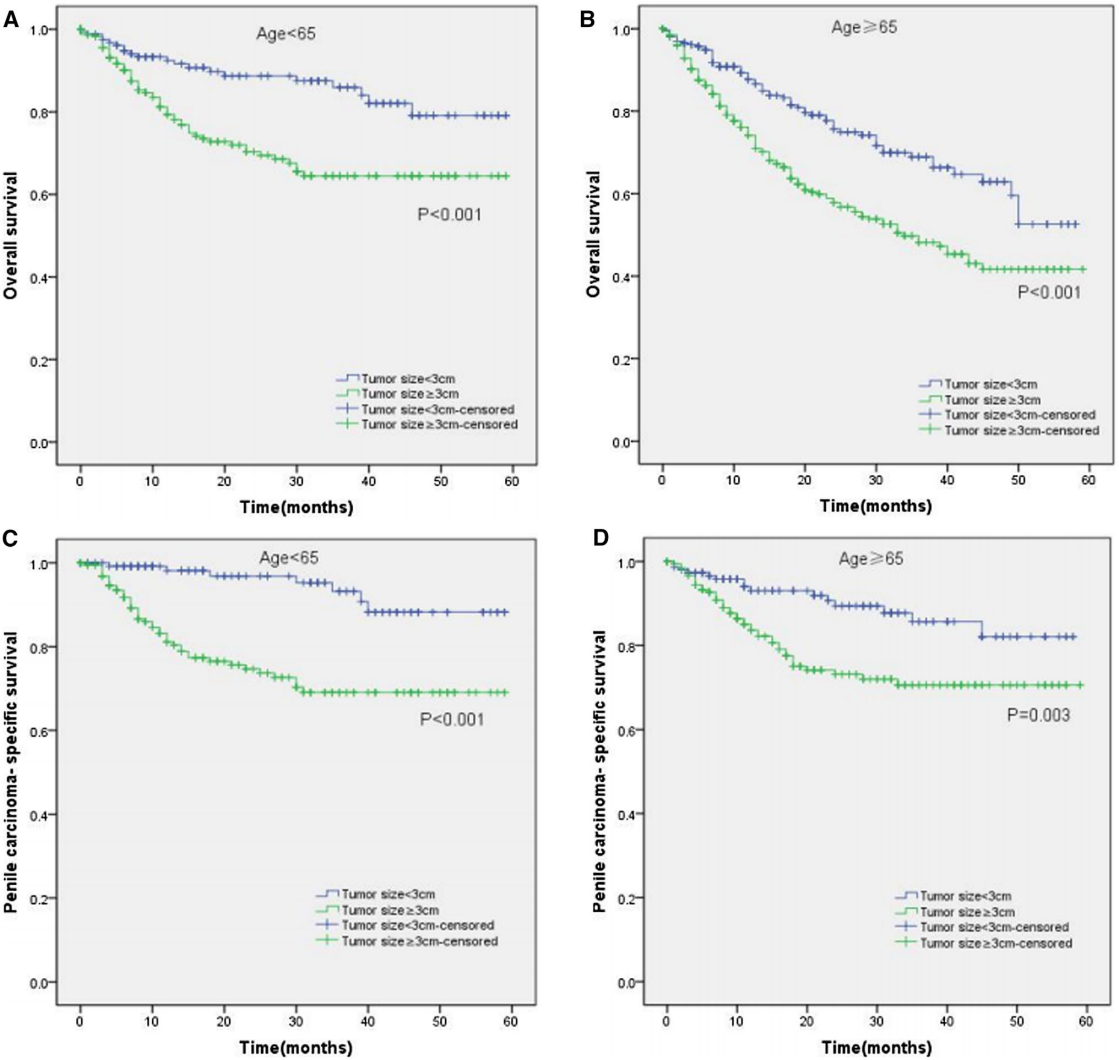
Kaplan–Meier analyses of overall survival (**A**, **B**) and penile carcinoma-specific survival (**C**, **D**) between age < 65 years old group and age ≥ 65 years old group in patients stratified by tumor size

**Fig. 4:**
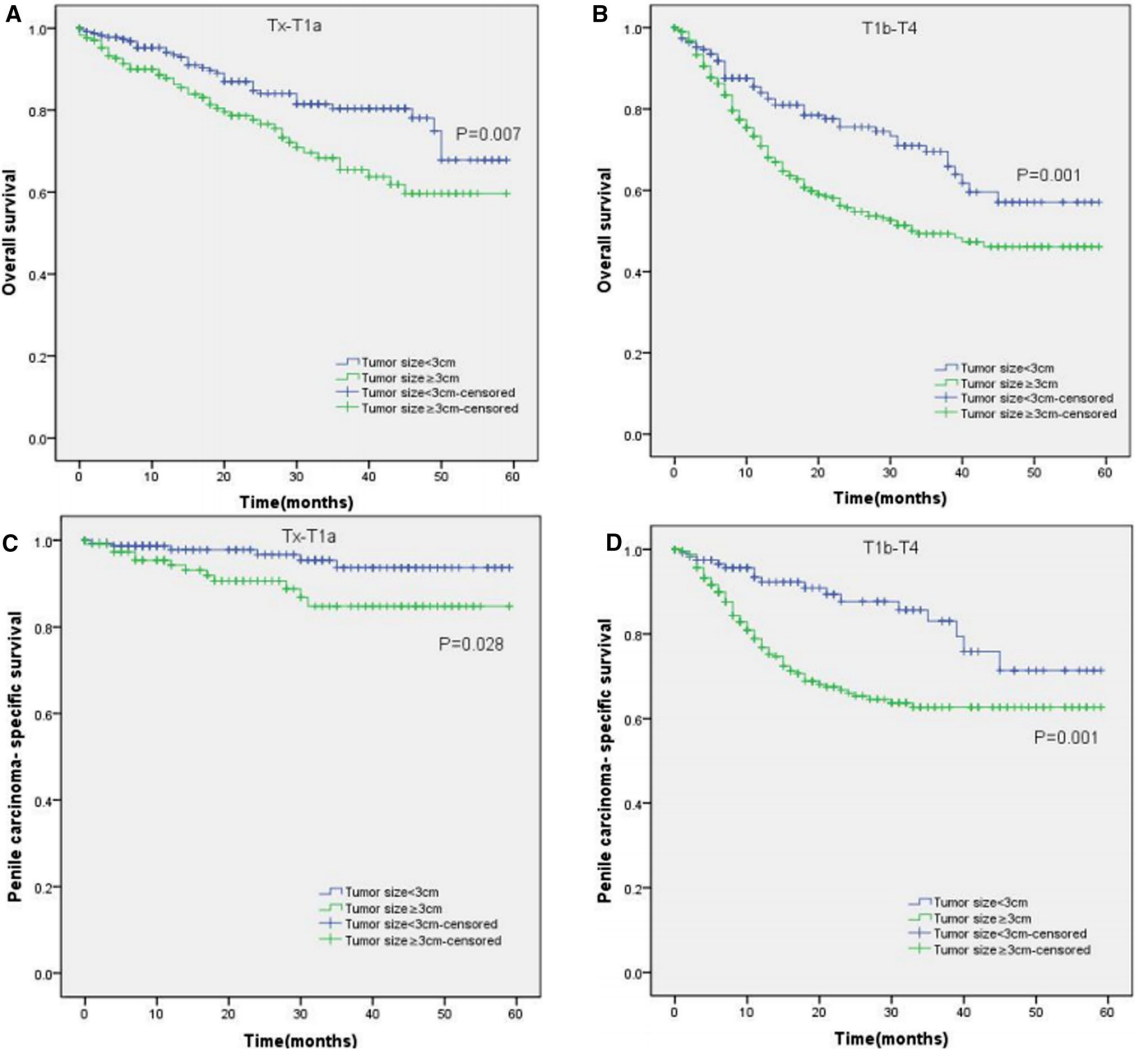
Kaplan–Meier analyses of overall survival (**A**, **B**) and penile carcinoma-specific survival (**C**, **D**) between Tx–T1a group and T1b–T4 group in patients stratified by tumor size

**Fig. 5:**
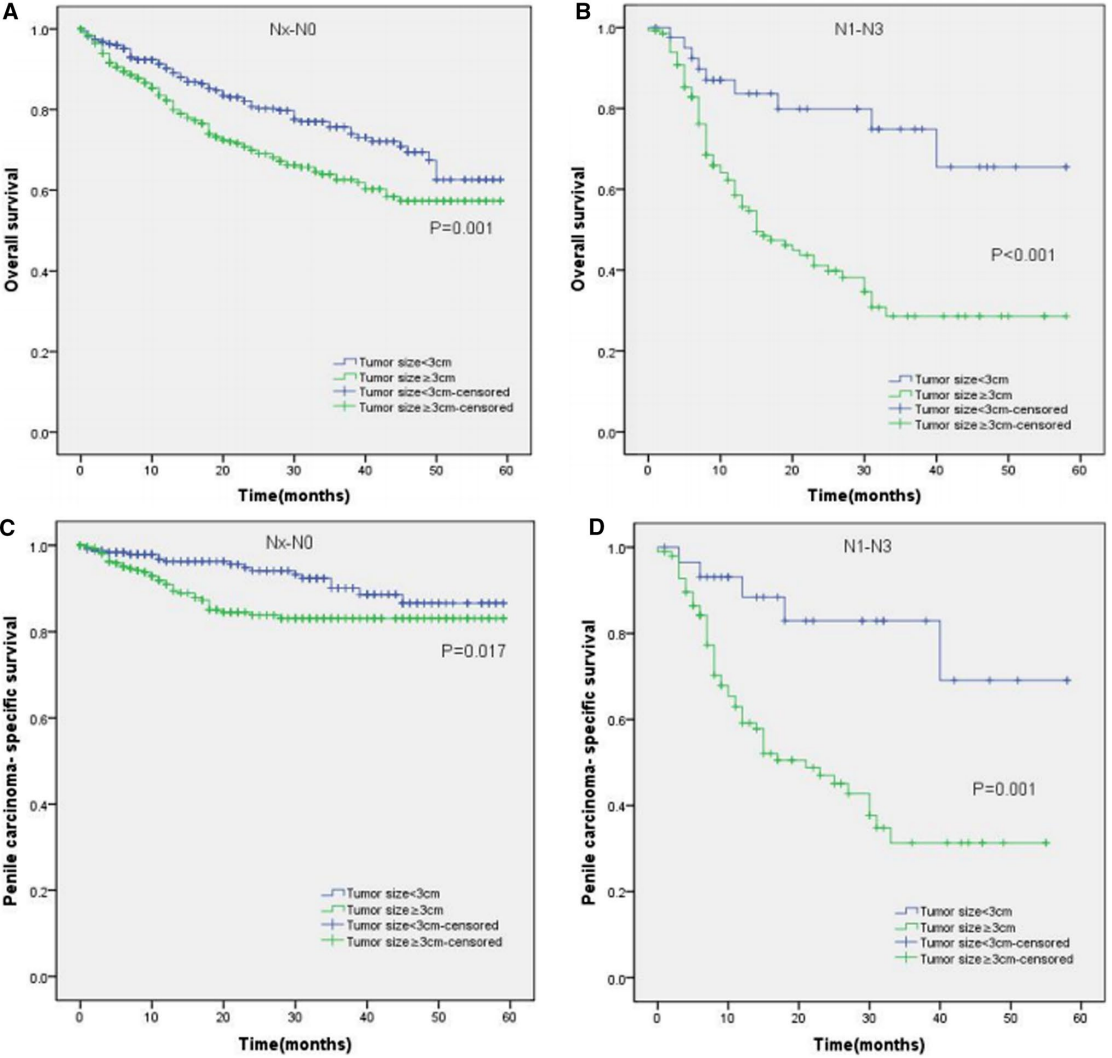
Kaplan–Meier analyses of overall survival (**A**, **B**) and penile carcinoma-specific survival (**C**, **D**) between Nx–N0 group and N1–N3 group in patients stratified by tumor size

**Fig. 6:**
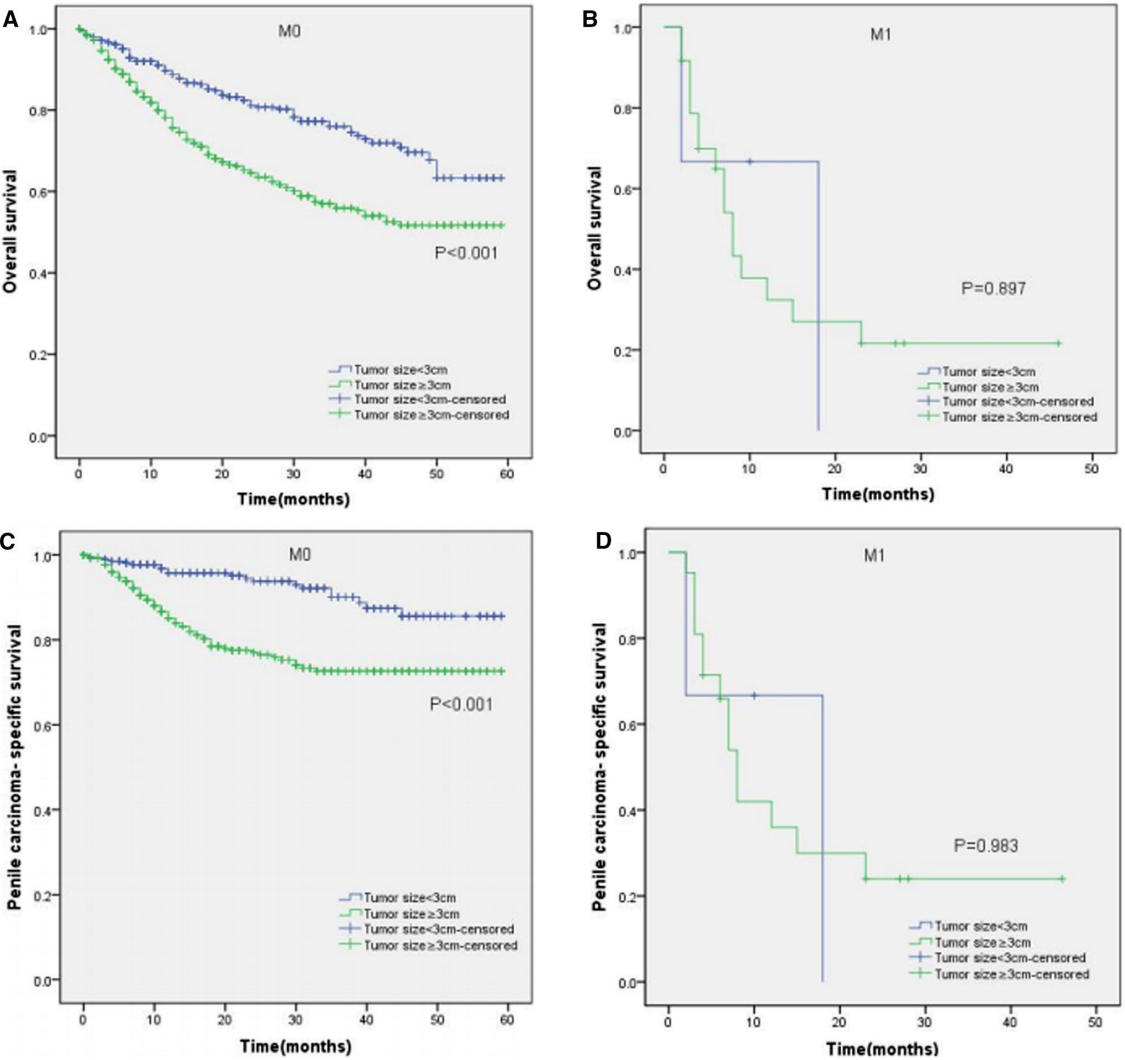
Kaplan–Meier analyses of overall survival (**A**, **B**) and penile carcinoma-specific survival (**C**, **D**) between M0 group and M1 group in patients stratified by tumor size

**Fig. 7:**
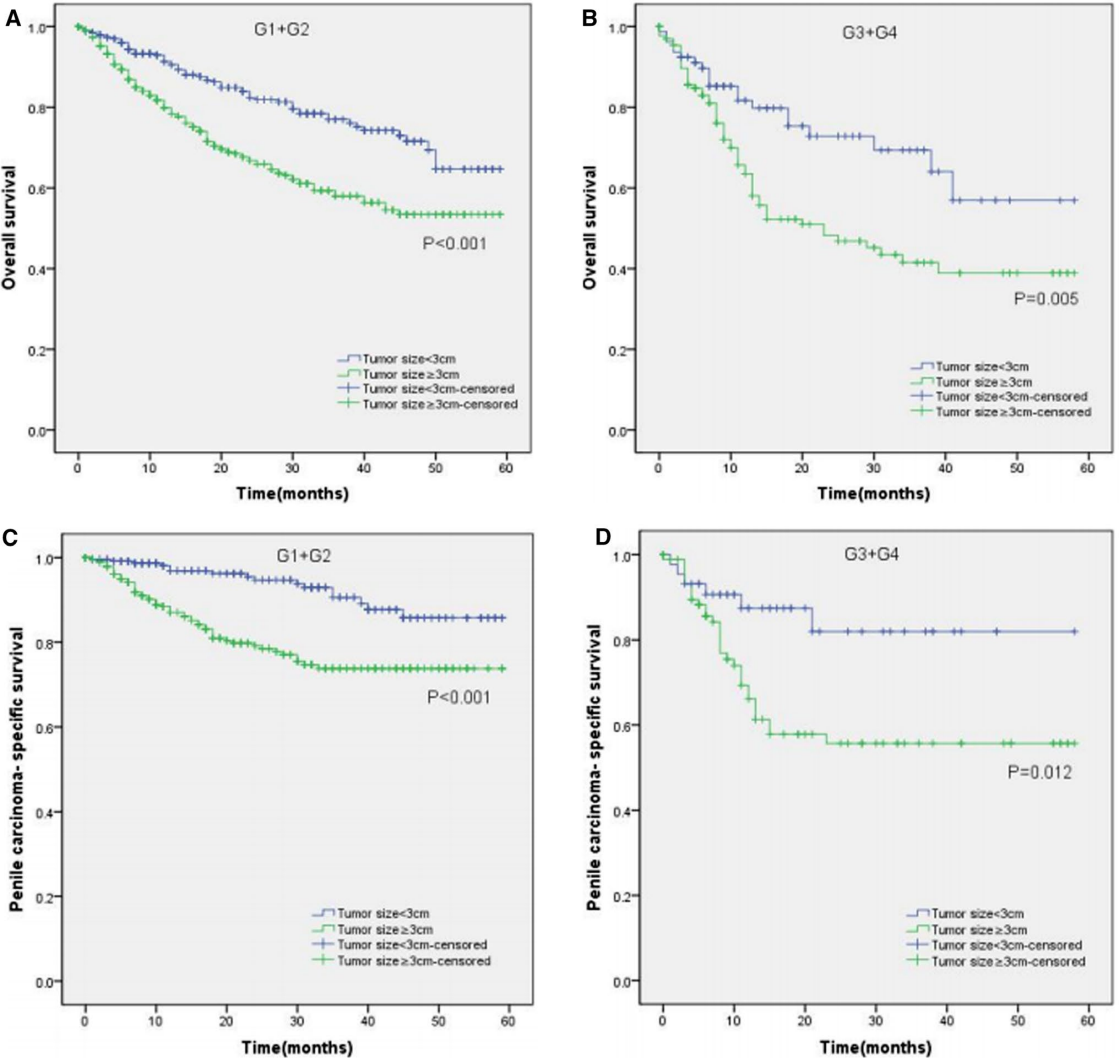
Kaplan–Meier analyses of overall survival (**A**, **B**) and penile carcinoma-specific survival (**C**, **D**) between G1 + G2 group and G3 + G4 group in patients stratified by tumor size

**Fig. 8:**
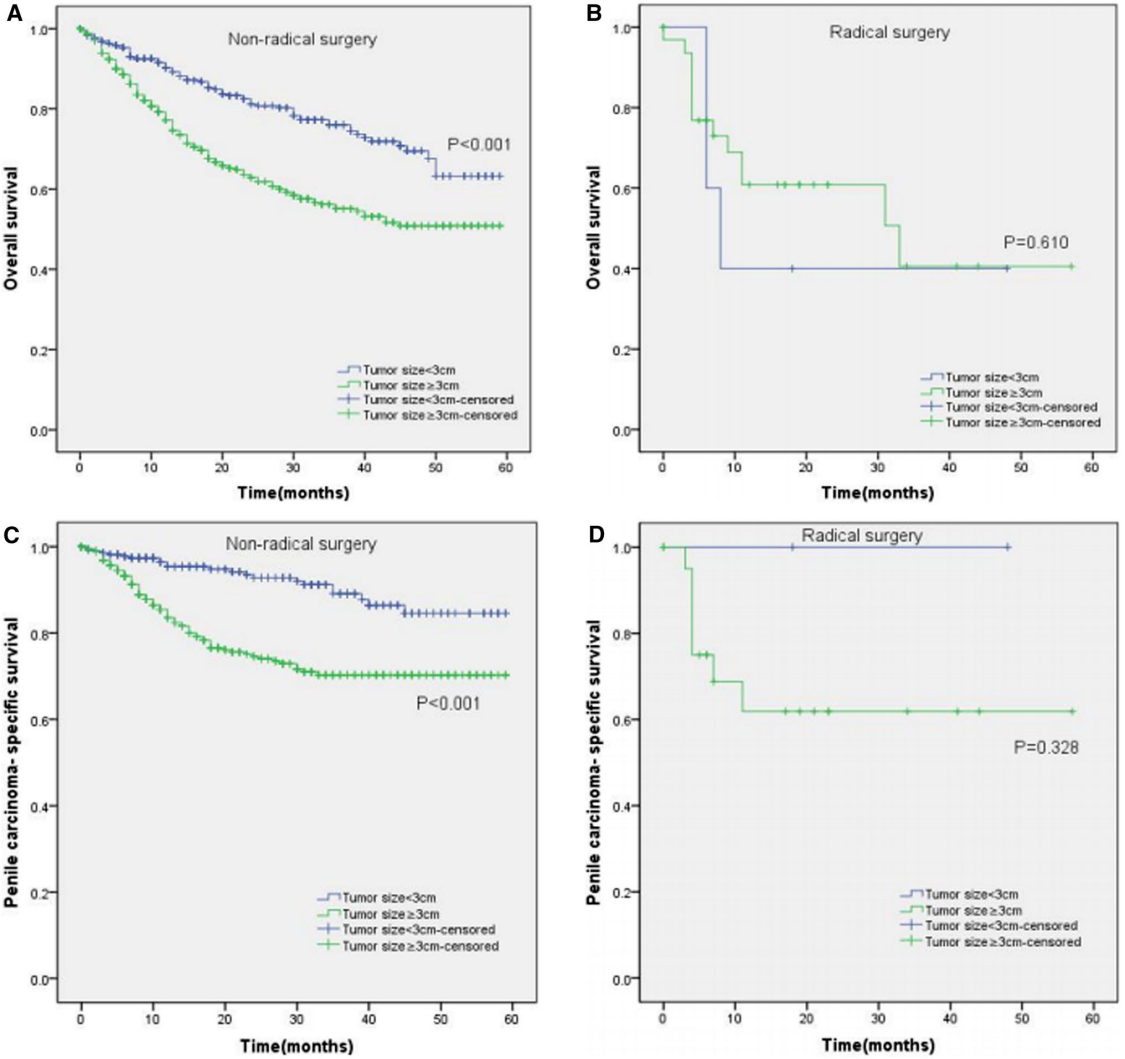
Kaplan–Meier analyses of overall survival (**A**, **B**) and penile carcinoma-specific survival (**C**, **D**) between non-radical surgery group and radical surgery group in patients stratified by tumor size

**Fig. 9:**
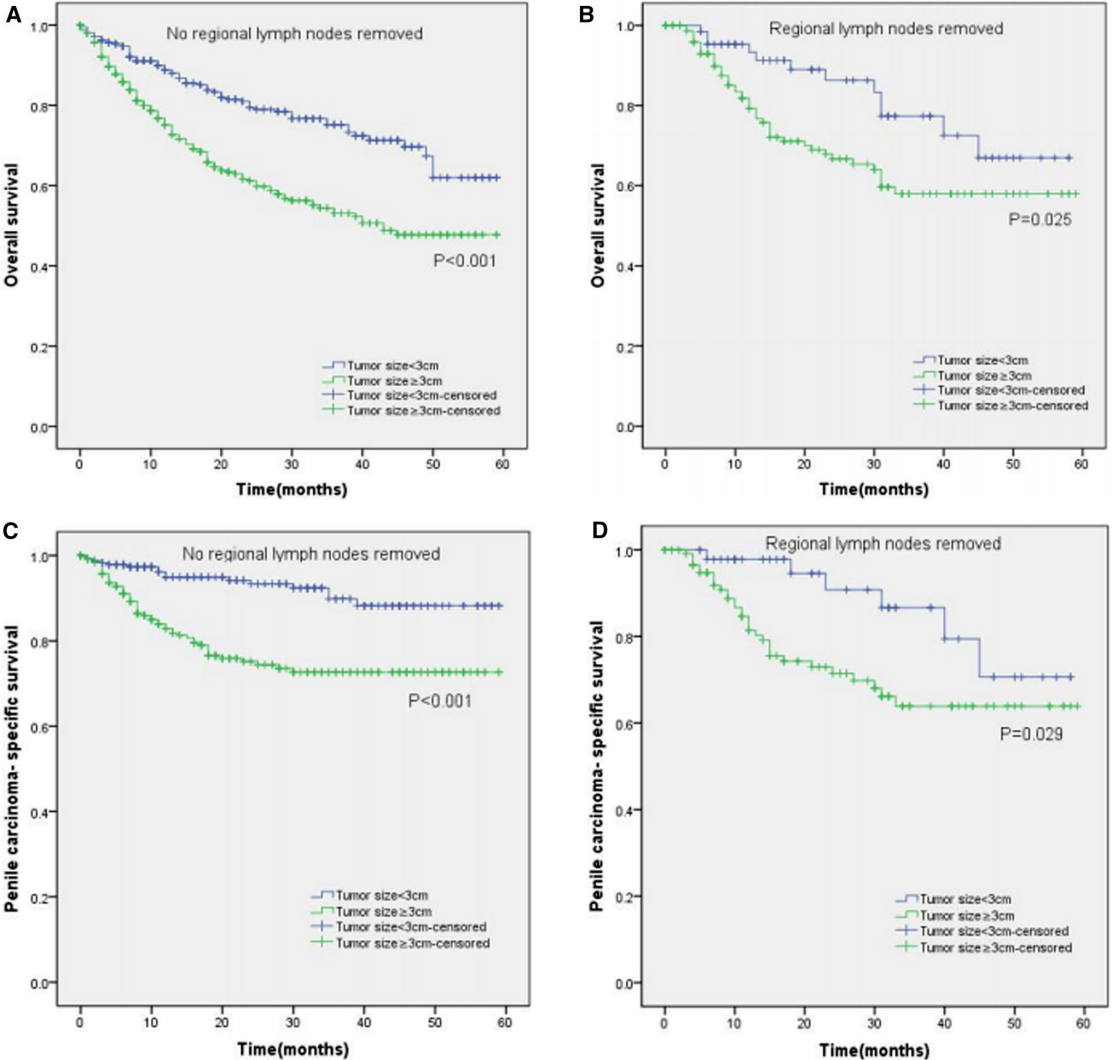
Kaplan–Meier analyses of overall survival (**A**, **B**) and penile carcinoma-specific survival (**C**, **D**) between no regional lymph nodes removed group and regional lymph nodes removed group in patients stratified by tumor size

Moreover, the KM curves of the stratified analyses showed that tumor size ≥ 3 cm was significantly associated with poorer OS and worse PCSS in patients with age < 65 years old (P < 0.001 for both, Fig. 3A, C), age ≥ 65 years old (P < 0.001, P = 0.003, Fig. 3B, D), Tx–T1a stage (P = 0.007, P = 0.028, Fig. 4A, C), T1b–T4 stage (P = 0.001 for both, Fig. 4B, D), Nx–N0 stage (P = 0.001, P = 0.017, Fig. 5A, C), N1–N3 stage (P < 0.001, P = 0.001, Fig. 5B, D), M0 stage (P < 0.001 for both, Fig. 6A, C), grade 1–2 (P < 0.001 for both, Fig. 7A, C), grade 3–4 (P = 0.005, P = 0.012, Fig. 7B, D), non-radical surgery (P < 0.001 for both, Fig. 8A, C), no regional lymph nodes removed (P < 0.001 for both, Fig. 9A, C), and regional lymph nodes removed (P = 0.025, P = 0.029, Fig. 9B, D).

As represented in Table 3, the multivariate Cox proportional hazards analyses revealed that tumor size in patients with SCCP was an independent predictor for both OS (HR 1.665, P < 0.001) and PCSS (HR 2.076, P = 0.003). Besides, T stage (HR 1.444, P = 0.016; HR 2.365, P = 0.003) and lymph node status (HR 1.809, P < 0.001; HR 4.595, P < 0.001) were also significant independent predictors for OS and PCSS. Notably, age (HR 1.933, P < 0.001) and distant metastasis (HR 2.934, P < 0.001) were significantly associated with OS, while regional lymph nodes removed (HR 0.513, P = 0.009) was associated with PCSS based on this model. However, grade (HR 1.197, P = 0.224; HR 1.483, P = 0.067) was not found to be significantly associated with OS or PCSS.

**Table 3:** Multivariate Cox regression analyses predicting overall survival and penis cancer-specific survival in 1001 patients with SCCP

Variables	Overall survival			Penis cancer-specific survival		
	Hazard ratio	95% CI	*P*	Hazard ratio	95% CI	*P*
Age	1.933	1.472–2.537	** < 0.001**	–	–	–
T stage	1.444	1.070–1.948	**0.016**	2.365	1.338–4.181	**0.003**
Lymph nodes status	1.809	1.359–2.408	** < 0.001**	4.595	2.776–7.604	** < 0.001**
Grade	1.197	0.896–1.598	0.224	1.483	0.973–2.261	0.067
Distant metastasis	2.934	1.748–4.922	** < 0.001**	1.760	0.918–3.375	0.089
Regional lymph nodes removed	–	–	**–**	0.513	0.310–0.848	**0.009**
Tumor size	1.665	1.276–2.172	** < 0.001**	2.076	1.278–3.373	**0.003**

*SCCP* squamous cell carcinoma of the penis, *CI* confidence intervals

Significant values in bold, “–” = no data

## Discussion

Squamous cell carcinoma of the penis is a rare but psychologically devastating malignant disease. Indeed, the rarity of this cancer limits the reliability and validity of reported prognostic factors and understanding of its associated risk factors [4]. In this study, we revealed that among the investigated clinicopathological characteristics that can be pre-or intraoperatively evaluated, large tumor size was a poor prognostic factor for SCCP. Therefore, a precise pre- and intra-operative assessment of the tumor size is crucial for improved survival of patients with SCCP. Given this finding, surgeons should take into consideration this important prognostic factor and individualized treatments accordingly.

The present study also indicated that advanced T stage (T1b–T4) was more common in SCCP patients with large tumors. According to the 2009 TNM clinical and pathological classification of penile cancer [19], the tumor invades subepithelial connective tissue with lymphovascular invasion in the TIb stage. Particularly in carcinomas, lymphovascular involvement often occurs before invasion [20] and usually forebodes advanced-stage disease [21]. Moreover, the lymphovascular invasion has been ascertained to be a significant risk factor for lymph node metastases [4, 22] and distant metastasis [7]. This evidence could explain why lymph node metastasis and distant metastasis were also common in men with large size of SCCP in this study. Similarly, Antonio et al. also suggested that the tumor size of SCCP with node metastasis tended to be larger [23]. Taken together, these findings suggested that large tumors were more likely to be invasive, as they exhibited an increased risk of being presented with advanced T-stage, lymph node metastasis, and distant metastasis. Accumulating studies have documented that lymph node metastasis and depth of invasion into penile anatomic levels are poor prognostic factors for SCCP [4, 12, 23, 24]. We have previously reported that distant metastasis was also significantly independently associated with poor OS and PCSS of men with SCCP [7]. Therefore, we hypothesized that SCCP patients with large tumors might exhibit poor prognosis due to the presence of the above-mentioned risk factors.

Furthermore, in our series, the data presented that the patients with large tumors exhibited significantly lower OS and PCSS than those with small tumors in Kaplan–Meier analyses and the stratified analyses. This finding confirmed our hypothesis and was consistent with a previous study conducted by Frederic et al. where they reported the adverse effect of large size of the penile cancer lesion on prognosis was evident from the 5-year survival rates in the 25 determinate cases, and the reason for the adverse effect might be the characteristics that increase the potential for metastasis, such as long duration of the lesion, high aggressiveness, and rapid growth [18].

A previous study by Antonio et al. reported no correlation between tumor size of SCCP and patients’ outcome [21]. However, the result of this study revealed that tumor size of SCCP was an independent prognostic factor for OS and PCSS in the Cox proportional hazards regression model, after adjusting for age, T stage, lymph nodes status, grade, distant metastasis, and regional lymph nodes removed. Surprisingly, to the best of our knowledge, the present study is the first to report that tumor size is a significant independent prognostic factor for SCCP.

In addition, this study also confirmed that regional lymph nodes removed and radical surgery were more frequently performed in SCCP patients with tumor size ≥ 3 cm than those with tumor size < 3 cm. In other words, patients with large size of SCCP are more possibly undergo lymphadenectomy and radical surgery. This study also realized that large-sized tumors are expected to be invasive. Similarly, a previous study reported that for invasive penile cancer, regional lymphadenectomy and amputation were preferred treatment strategies [25].

This study with a large sample size was a population-based assessment, which significantly reduced differences and biases; besides, large sample size was the major advantage of this study compared to previously conducted studies. However, there were several limitations to this study. First, the results of this study were all analyzed retrospectively, and it is highly unlikely that large prospective studies will be organized to validate the prognostic significance of tumor size in patients with SCCP. Second, we didn’t have access to the medical records of all patients and therefore had to rely on the data of cases recorded in the SEER database. Third, The SEER database does not provide information on the comorbidities that may significantly affect survival rates. Fourth, the population in the SEER database is from the United States, so our findings may not apply to other populations.

## Conclusions

In conclusion, this SEER-based study revealed that large tumor size was significantly associated with increased risk of SCCP patients being presented with advanced T stage, lymph node metastasis, and distant metastasis. Also, the patients with large tumors exhibited an inferior OS and PCSS than those with small tumors. Besides, tumor size was an independent prognostic factor for OS and PCSS. Therefore, clinical assessment of tumor size as a crucial prognostic factor might be highly beneficial for early intervention in patients with SCCP.

## Data Availability

The web address of SEER database is https://seer.cancer.gov/, and there are no restrictions on the use of this database by non-academics.
